# Melatonin Treatment Improves Postharvest Preservation and Resistance of Guava Fruit (*Psidium guajava* L.)

**DOI:** 10.3390/foods11030262

**Published:** 2022-01-19

**Authors:** Silin Fan, Tiantian Xiong, Qiumei Lei, Qinqin Tan, Jiahui Cai, Zunyang Song, Meiyan Yang, Weixin Chen, Xueping Li, Xiaoyang Zhu

**Affiliations:** 1 Guangdong Provincial Key Laboratory of Postharvest Science of Fruits and Vegetables/Engineering Research Center for Postharvest Technology of Horticultural Crops in South China, Ministry of Education, College of Horticulture, South China Agricultural University, Guangzhou 510642, China; 1575644390@foxmail.com (S.F.); 1198960312@foxmail.com (Q.L.); 1621251540@foxmail.com (Q.T.); jh.cai_chn@outlook.com (J.C.); songzunyang@163.com (Z.S.); ymy@scau.edu.cn (M.Y.); wxchen@scau.edu.cn (W.C.); lxp88@scau.edu.cn (X.L.); 2 Key Laboratory of Ecology and Environmental Science in Guangdong Higher Education, School of Life Science, South China Normal University, Guangzhou 510631, China; xiongtiantian@m.scnu.edu.cn

**Keywords:** ripening, reactive oxygen species, enzymatic activities, anthracnose, shelf-life

## Abstract

Guava fruit has a short postharvest shelf life at room temperature. Melatonin is widely used for preservation of various postharvest fruit and vegetables. In this study, an optimal melatonin treatment (600 μmol·L^−1^, 2 h) was identified, which effectively delayed fruit softening and reduced the incidence of anthracnose on guava fruit. Melatonin effectively enhanced the antioxidant capacity and reduced the oxidative damage to the fruit by reducing the contents of superoxide anions, hydrogen peroxide and malondialdehyde; improving the overall antioxidant capacity and enhancing the enzymatic antioxidants and non-enzymatic antioxidants. Melatonin significantly enhanced the activities of catalase, superoxide dismutase, ascorbate peroxidase and glutathione reductase. The contents of total flavonoids and ascorbic acid were maintained by melatonin. This treatment also enhanced the defense-related enzymatic activities of chitinase and phenylpropanoid pathway enzymes, including phenylalanine ammonia lyase and 4-coumaric acid-CoA-ligase. The activities of lipase, lipoxygenase and phospholipase D related to lipid metabolism were repressed by melatonin. These results showed that exogenous melatonin can maintain the quality of guava fruit and enhance its resistance to disease by improving the antioxidant and defense systems of the fruit.

## 1. Introduction

Guava (*Psidium guajava* L.) is native to tropical America from Mexico to Peru, and now a commercially important fruit and medicinal plant in many tropical and subtropical countries [1]. Countries producing and consuming most guava include India, Mexico, Pakistan, Brazil, Egypt, Thailand, Colombia [1], as well as China in recent years. The significance of guava fruit may be owing to its high production, remarkable nutritional properties and enchanting taste, since it contains five times as much ascorbic acid as citrus fruit and also contains various essential bioactive compounds [2]. Guava has high levels of dietary fiber, pectin, antioxidants, vitamins and mineral content compared with other fruit [2,3]. However, guava fruit with thin peel and soft texture are highly susceptible to chilling stress, mechanical injury and pathogens. Therefore, it has a short postharvest life, which severely limits its consumption and distribution to domestic markets [4]. The fruit soften rapidly, so they tend to bruise and over-ripen, which causes severe losses during the postharvest period. To date, various postharvest handling techniques have been utilized to regulate the ripening of fruit and reduce losses from postharvest physiological disorders and diseases. Low temperature storage is a well-known and common method to extend the storage period of guava fruit. However, storage under 10 °C can cause serious chilling injury (CI) to guava fruit, including the browning or discoloration of the peel and pulp, abnormal ripening and an increase in the incidence of anthracnose caused by *Colletotrichum gloeosporioides* [5]. Anthracnose is one of the principal postharvest diseases of several tropical and subtropical fruit, including papayas, mangoes, bananas, apples, passion fruit, guavas, grapes and citrus [6]. As the most common fungal disease of guava fruit (*Lim and Manicom*), anthracnose causes severe postharvest losses of these fruit [1]. The anthracnose disease commonly infects quiescently, initiating infection during flowering and remaining latent until it is activated by ripening during fruit storage, transport and shelf life [6,7]. Different technologies have been explored to reduce the incidence of anthracnose in guava fruit. 1-methylcyclopropene (1-MCP), an effective inhibitor of ethylene, is widely used to extend the shelf life of fruit. 1-MCP has a pronounced effect on fruit ripening and significantly maintains high fruit firmness, maintains fruit quality and reduces the incidence of decay [4]. Other commonly used techniques, such as edible coating treatments, modified atmosphere, and controlled atmosphere storage, ascorbic acid treatment, gamma irradiation, fungicides, calcium chloride and nitric oxide, can reduce the fruit peel browning index, maintain the fruit quality and extend the shelf life of guava fruit [1,3,8,9,10,11,12,13].

Melatonin (N-acetyl-5-methoxytryptamine) is a multifunctional signaling molecule that is ubiquitously distributed in various plant species [14]. Melatonin is involved in various physiological activities in plants, including seed and root development [15], plant growth and flowering [16], fruit development [17], fruit ripening [18], senescence [19] and biotic and abiotic stress responses [20,21]. As a safe and nontoxic substance, melatonin is widely studied in postharvest preservation for its role in improving the storage life and quality of fruit and vegetables, as well as enhancing crop production and ensuring food safety in an environmentally friendly manner. Various studies have proven that the application of exogenous melatonin significantly delayed the postharvest ripening of some fruit, such as apples [22], sweet cherries [23], bananas [19,24], pears [25], peaches [26] and mangoes [27]. Exogenous treatment with melatonin can also enhance the resistance to disease and reduce the incidence of decay of various fruit during postharvest storage, including litchis [28], strawberries [29], kiwifruits [30] and peaches [26]. 

This study aims to explore the effect of melatonin on guava fruit ripening and quality during the postharvest storage periods. The results showed that treatment with melatonin significantly delayed fruit ripening and repressed the incidence of anthracnose during the postharvest periods. In particular, treatment with melatonin effectively enhanced the antioxidant capacity and reduced the oxidative damage to the fruit. These results provide technical and theoretical guidance for the practical application of melatonin in the postharvest stage of guava fruit during storage and marketing.

## 2. Materials and Methods

### 2.1. Plant Materials and Treatments

Guava fruit (*Psidium guajava* L. ‘Zhenzhu’) at the commercial stage (mature-green) were harvested from a local commercial plantation of Baoshen Farm, Nansha district, Guangzhou, South China, and immediately transported to the laboratory. Fruit without blemishes that were uniformly sized were selected, cleaned and dipped in a 0.2% solution of sodium hypochlorite solution for 10 min. After air-drying under the room temperature (22 ± 2 °C) for 2 h (h), selected fruit were soaked in different concentrations of melatonin (Shanghai Shenggong Biology Limited Company, Shanghai, China) for 2 h. The melatonin was dissolved in ethanol and then diluted to 0 μmol·L^−1^, 100 μmol·L^−1^, 400 μmol·L^−1^, and 600 μmol·L^−1^ for treatment. After the fruit were removed from the solution and air-dried at room temperature (22 ± 2 °C), they were placed into unsealed plastic bags (0.02 mm thick), and stored at 25 ± 1 °C with 70~80% relative humidity. Three independent biological replicates were performed for each treatment, where each replicate included 45 fruits. The fruit were periodically evaluated by ripening indicators. Samples were taken at 0, 3, 6, 8, 11, 13 and 15 days after treatment.

### 2.2. Fruit Firmness, Respiration Rate and Color Index

Fruit firmness was measured using an Instron Harness Tester 5542 (Instron, Norwood, NJ, USA) as described by Zhu et al. [31]. The measurements were conducted on five points of each fruit, and the results were expressed in Newtons.

The respiration rate was determined as described by Li et al. [32]. Briefly, the fruit were placed in a 2.5 L plastic container after weighing and held for 2 h at 25 °C. Next, 1 mL of headspace gas sample was extracted and measured using a gas chromatograph (G3900, Hitachi, Ltd., Tokyo, Japan). The respiration rate was expressed by the rates of CO_2_ production, which were expressed as milligram per kilogram per hour.

Fruit color was measured as described by Li et al. [19] with minor modifications. Five points around the equatorial region on each fruit were selected for measurement using a Chromameter-2 reflectance colorimeter (Minolta, Osaka, Japan) equipped with a CR-300 measuring head. The results were recorded as lightness (L*), hue angle (h°), and chroma (C*).

### 2.3. Fruit Disease Evaluation

The incidence of disease and severity on the fruit were observed periodically as described by Pandey et al. [33] with minor modifications. The disease severity was recorded daily by measuring the lesion area on each fruit as compared to the total fruit surface area using a scale as follows: 0 = no lesion, 1 = 1–10%, 2 = 11–20%, 3 = 21–30%, 4 = 31–40%, 5 = 41–50%, 6 = 51–60%, 7 = 61–70%, 8 = 71–80%, 9 = 81–90%, and 10 = 91–100% of the area of the fruit was diseased.

The incidence of disease was calculated as the ratio of the number of diseased fruit to the total number of fruit [4].

### 2.4. Determination of Reactive Oxygen Species (ROS) and Malondialdehyde Contents

The contents of hydrogen peroxide (H_2_O_2_), superoxide radicals (O^2−^) and malondialdehyde (MDA) were measured by UV-Vis spectrophotometry using kits (No. H2O2-2-Y, SA-2-G, and MDA-2-Y; Suzhou Keming Biological Company, Suzhou, China), according to the manufacturer’s instructions. The absorbance was measured at 530 nm and calculated for the O^2−^ content with a unit expressed as nmol·g^−^^1^. The absorbance at 415 nm was recorded for the content of H_2_O_2_ with a unit expressed as μmol·mL^−^^1^. The content of MDA was determined by measuring the absorbance at 532 nm, and the unit was expressed as nmol·g^−^^1^.

### 2.5. Enzyme Activities and Metabolites Content Measurement

#### 2.5.1. The Antioxidative Enzyme Activities and Antioxidant Content Detection

The activities of catalase (CAT), superoxide dismutase (SOD), glutathione reductase (GR), ascorbate peroxidase (APX) and the total antioxidant capacity (T-AOC) were determined by UV-Vis spectrophotometry using different biochemical kits (No. SOD-2-W, CAT-2-W, GR-2-W, APX-2-W, ASA-1-W, and FRAP-2-G; Suzhou Keming Biological Company), according to the manufacturer’s instructions. The absorbance from the activity of SOD was 450 nm, which was expressed as U·g^−^^1^, and that of CAT was 240 nm, which was expressed as nmol/min^−^^1^/g^−^^1^. The absorbance value at 340 nm was recorded, and the unit was expressed as nmol·min^−^^1^·g^−^^1^ for GR activity. For APX activity, the absorbance was recorded at 290 nm, and the unit was expressed as μmol·min^−^^1^·g^−^^1^. The absorbance was recorded at 593 nm for T-AOC and the unit expressed as μmol Trolox^−^^1^·g^−^^1^.

#### 2.5.2. The Determination of Flavonoids and Ascorbic Acid Contents

The contents of total flavonoids and reduced ascorbic acid (ASA) were determined using a total flavonoid content kit and an ASA content kit (No. LHT-2-G, No. ASA-1-W; Suzhou Keming Biological Company) according to the manufacturer’s instructions. The absorbance was measured at 510 nm and the unit expressed in mg·g^−1^ for the total flavonoid content. The content of ASA was measured at 420 nm and expressed as μg·g^−^^1^.

#### 2.5.3. Determination of the Activities of Enzymes Related to Phenylpropanoid Metabolism

The activities of three key enzymes involved in phenylpropanoid metabolism were determined, including 4-coumaric acid: coenzyme A ligase (4CL), cinnamic acid-4-hydroxylase activity (C4H) and phenylalanine ammonia lyase (PAL). The activities of 4CL and C4H were determined using an enzyme activity kit (No. G1003W and G1001W; Suzhou Geruisi Biological Company, Suzhou, China). For 4CL, the rate of production of 4-coumaric acid CoA at 333 nm was measured to determine the activity of 4CL in nmol·min^−^^1^·g^−^^1^. The absorbance was read at 340 nm for C4H and expressed as nmol·min^−^^1^·g^−^^1^.

The activity of PAL was measured at 290 nm and expressed as U·g^−^^1^ using a kit according to the manufacturer’s instructions (No. PAL-2-Y; Suzhou Keming Biological Company). 

#### 2.5.4. Determination of Enzyme Activities Related to the Lipid Metabolism of Fruit Membranes 

The activities of lipase (LPS), lipoxygenase (LOX) and phospholipase D (PLD) were determined using kits according to the manufacturer’s instructions (No. G0902F, G0906F, and G0925F; Suzhou Geruisi Biotechnology Co. Ltd.). The activity of LPS was measured at 405 nm and expressed as μmol·min^−^^1^·g^−^^1^. The activity of LOX was assayed at 234 nm and expressed as U·g^−^^1^. For PLD, the absorption was measured at 500 nm and expressed as nmol·min^−^^1^·g^−^^1^.

#### 2.5.5. Determination of Enzyme Activities Related to Fruit Resistance

The activity of chitinase (CHI) activity was determined using a CHI content micro-assay kit (No. GO546W; Suzhou Geruisi Biological Company). The absorption at 585 nm was measured and expressed as mg·h^−^^1^·g^−^^1^.

The β-1,3-glucanase (β-1,3-GA) activity was assayed using a β-1,3-GA activity kit (No. GA-1-Y; Suzhou Keming Biological Company). The absorption at 550 nm was measured and the unit expressed as mg·h^−^^1^·g^−^^1^.

### 2.6. Statistical Analysis

The data were analyzed using SPSS 17.0 (SPSS Inc., Chicago, IL, USA) and Microsoft Excel 2019 (Redmond, WA, USA) to calculate the means and standard deviation (SD). The figures were plotted using SigmaPlot 12.0 (Systat, Chicago, IL, USA). Each experiment was conducted using three biological repetitions. Significant differences between treatment groups were determined using Duncan’s multiple range test and least significant difference (LSD) (*p* < 0.05). All the results are expressed as the means ± SD (standard deviation) (Supplemental Table S1).

## 3. Results

### 3.1. The Effects of Melatonin on Fruit Ripening

**Figure 1:**
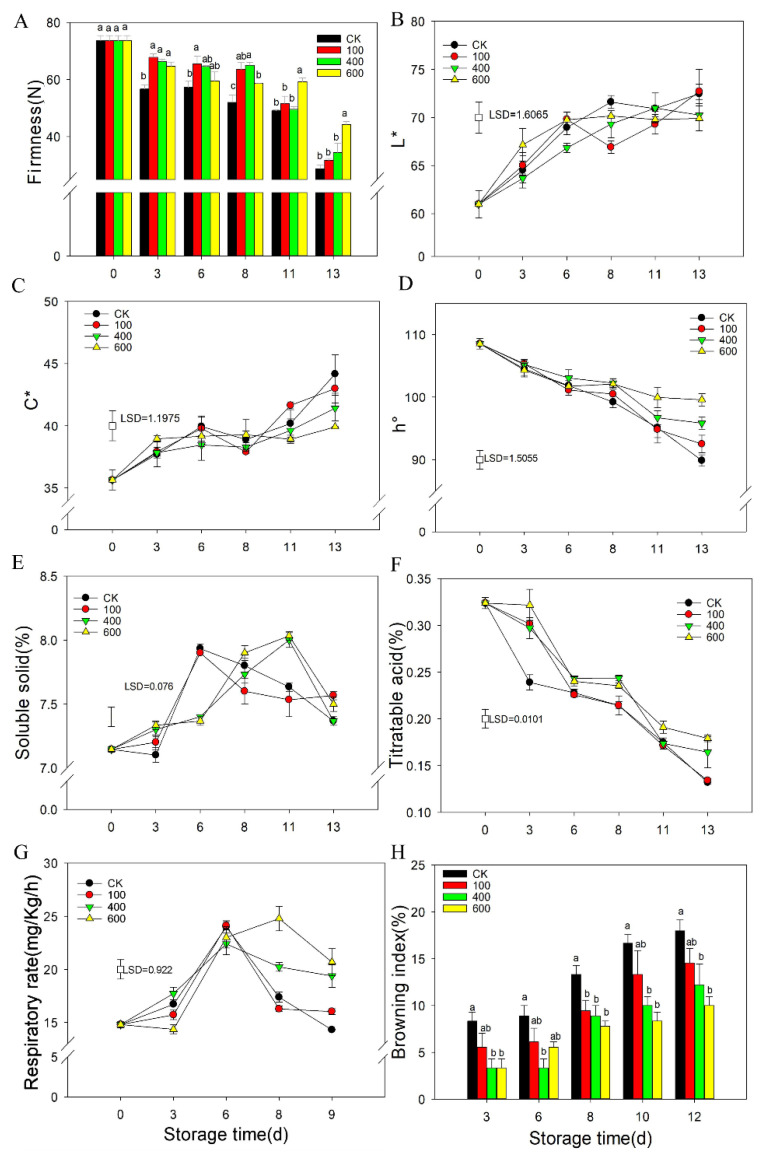
Effect of melatonin treatments on fruit physiology including the hardness, fruit color changes, soluble solid content, titratable acid, respiration rate and browning index. (**A**), the effect of melatonin treatment on the hardness of guava fruit; (**B**–**D**), the effect of melatonin treatment on the chromaticity L*, C*, h° of guava fruit; (**E**), the effect of melatonin treatment on soluble solid content; (**F**), the effect of melatonin treatment on the titratable acid content; (**G**), the effect of melatonin treatment on the respiration rate of guava fruit; (**H**), the effect of melatonin treatment on the browning index of guava fruit. Each data point represents the mean ± S.D. (n = 3). Different letters indicated the significant differences at the 5% level. Least significant differences (LSDs) were calculated to compare significant effects at the 5% level. Different letters indicate significant differences between groups (*p* < 0.05). CK: control group; 100: 100 μmol·L^−1^ melatonin treatment group; 400: 400 μmol·L^−1^ melatonin treatment group; 600: 600 μmol·L^−1^ melatonin treatment group.

Fruit firmness is one of the most important indicators for fruit ripening. As shown in Figure 1A, the fruit firmness decreased gradually with fruit storage under controlled conditions. All the melatonin treatments delayed the decrease in fruit firmness and maintained higher fruit firmness compared with the control (CK) group, particularly for the 600 μmol·L^−1^ treatment, which had significantly higher firmness than the other treatments at the end of storage (Figure 1A). The color of the peel was primarily evaluated by L*, C*, and h° values, which indicate the brightness, color saturation and the hue angle of the peel, respectively. As shown in Figure 1B, the L* value gradually increased with fruit ripening for the control group. No significant difference was observed between the melatonin treatment groups and the control group for the L* value (Figure 1B). The C* value of fruit increased with fruit ripening, and no significant difference was observed between the different groups (Figure 1C). The h° value gradually decreased with fruit ripening. The melatonin treatments delayed the decrease in h° value, which resulted in fruit with a higher h° value than the control during later storage (Figure 1D).

The soluble solid content of the fruit increased with ripening and reached a peak at six days and then decreased gradually in the control group. No significant difference was observed between the 100 μmol·L^−1^ melatonin treatment group and the control group. However, the 400 μmol·L^−1^ and 600 μmol·L^−1^ melatonin treatments delayed the soluble solid content peak by approximately five days and maintained a higher content during later storage (Figure 1E). The titratable acid content decreased with fruit ripening, and the melatonin treatments reduced the decrease more than the control group (Figure 1F). Fruit respiration increased with ripening and peaked on day six before gradually decreasing (Figure 1F). The 600 μmol·L^−1^ melatonin treatment repressed the respiration rate at day three but enhanced it during the later storage. The 400 μmol·L^−1^ melatonin treatment also enhanced the respiration rate during the later storage with no difference during the first six days. No significant significance was observed between the 100 μmol·L^−1^ melatonin treatment and the control group during the entire storage period (Figure 1G). Fruit showed browning symptoms from day three of storage, and the browning index increased with fruit storage under the control conditions. The melatonin treatments reduced the browning index, particularly for the 600 μmol·L^−1^ melatonin treatment, except for the 100 μmol·L^−1^ melatonin treatment (Figure 1H).

### 3.2. Effects of Different Concentrations of Melatonin on the Development of Fruit Disease 

**Figure 2:**
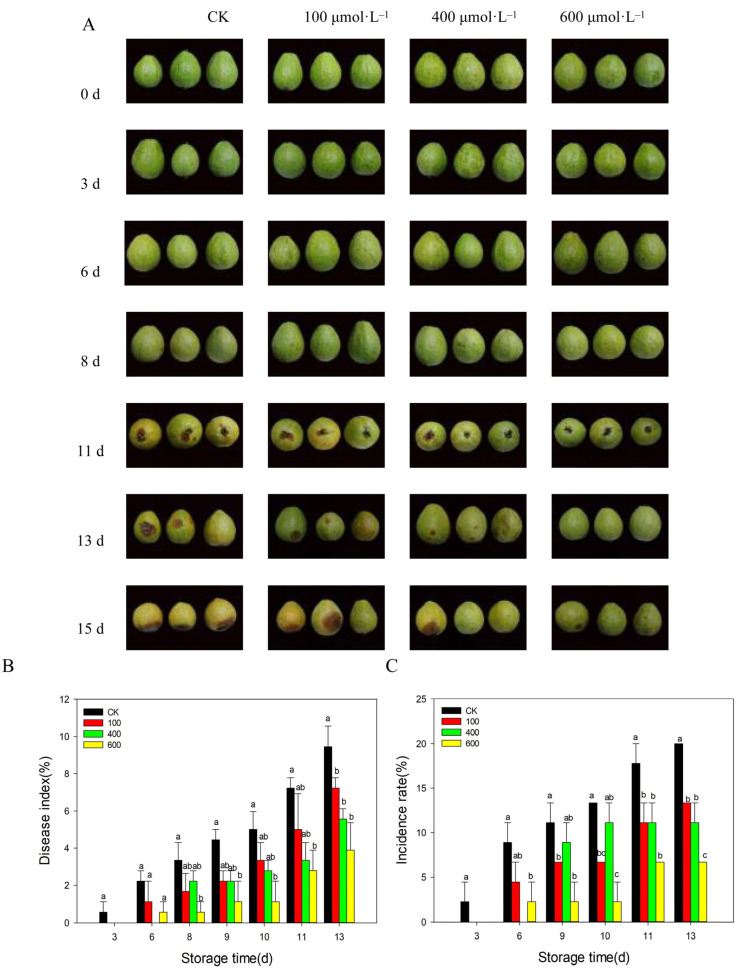
Effects of different concentrations of melatonin on fruit disease incidence. (**A**) Photos of guava fruit under different melatonin treatments; (**B**) fruit disease index; (**C**) fruit disease incidence. Guava fruit were stored at room temperature (25 ± 1 ℃) after treatment. Each data point represents the mean ± S.D. (n = 3). Different letters indicated the significant differences at the 5% level. CK: control group; 100: 100 μmol·L^−1^ melatonin treatment group; 400: 400 μmol·L^−1^ melatonin treatment group; 600: 600 μmol·L^−1^ melatonin treatment group.

Anthracnose is the most common disease for guava during the postharvest period. As shown in Figure 2, serious disease symptoms on the control fruit were observed on day 11 after harvest. Melatonin treatments significantly reduced and delayed the development of disease. This was particularly true for the 600 μmol·L^−1^ melatonin treatment in which disease was only observed after 15 days of storage (Figure 2A). As shown in Figure 2B, the disease index increased rapidly with fruit ripening. Melatonin treatments inhibited the development of disease index, which was significantly lower than that of the control fruit during the later storage, particularly for the 600 μmol·L^−1^ melatonin treatment (Figure 2B). Similar results were observed for the disease incidence (Figure 2C), and treatment with 600 μmol·L^−1^ melatonin resulted in the lowest disease incidence rate of all of the groups. 

Additionally, the Principal Component Analysis (PCA) also identified that both concentrations and storage times of melatonin treatments have significant effects on the overall variation of physiological/biochemical features in guava fruit (Figure S1).

### 3.3. Effects of Melatonin on the Metabolism of Reactive Oxygen Species 

**Figure 3:**
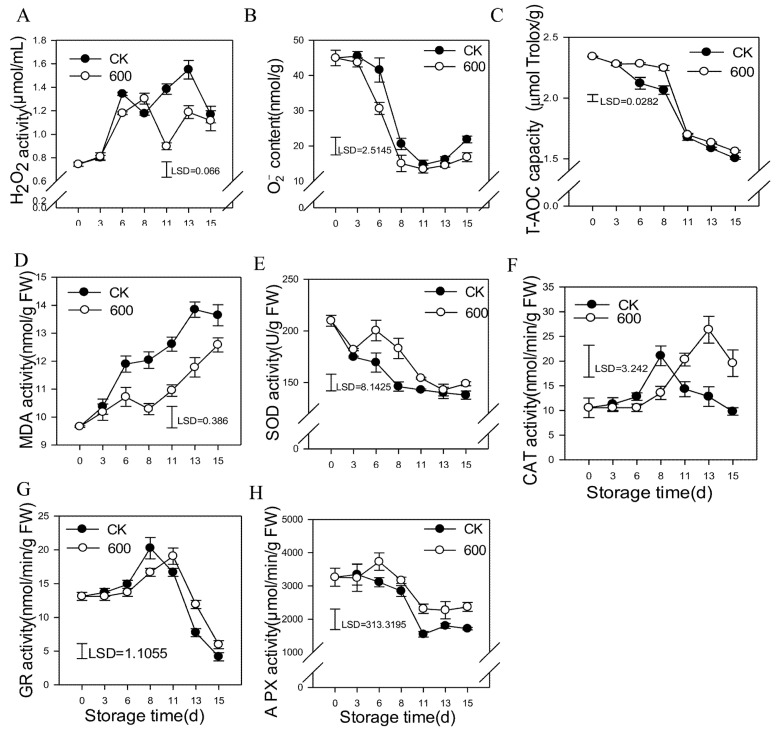
Effects of melatonin on the metabolism of reactive oxygen species in guava fruit. (**A**) the effect of melatonin treatment on H_2_O_2_ content; (**B**) the effect of melatonin treatment on O^2−^ content. (**C**) the effect of melatonin treatment on T-AOC capacity; (**D**) the effect of melatonin treatment on the MDA content; (**E**–**H)** the effect of melatonin treatment on the activities of SOD (**E**), CAT (**F**), GR (**G**) and APX (**H**). Each data point represents the mean ± S.D. (n = 3). Least significant differences (LSDs) were calculated to compare significant effects at the 5% level. CK: control group; 600: 600 μmol·L^−1^ melatonin treatment group.

Reactive oxygen species (ROS) are important in fruit ripening and stress responses. Hydrogen peroxide (H_2_O_2_) and O^2−^ are important ROS in plants when they are subjected to adverse conditions. As shown in Figure 3, the content of H_2_O_2_ gradually increased with fruit ripening and decreased at the end of storage in the control group. Treatment with melatonin reduced the content of H_2_O_2_ during the later storage resulting in significantly lower levels than those in the control group (Figure 3A). The content of O^2−^ gradually decreased as the fruit ripened and increased slightly at the end of storage in the control group. The melatonin treatment reduced the content of O^2−^ during the entire storage period (Figure 3B). Various antioxidants and antioxidant enzymes in guava fruit constitute the total antioxidant capacity (T-AOC). The T-AOC capacity decreased gradually with fruit storage, and treatment with melatonin maintained a high capacity of T-AOC during the first eight days of storage. However, there was no significant difference compared with the control group during the later storage period (Figure 3C). MDA is the final product of cell lipid peroxidation. As shown in Figure 3D, the content of MDA increased gradually in the control group as the fruit ripened (Figure 3C). Treatment with melatonin significantly reduced the content of MDA during the whole storage period, which was significantly lower than that of the control group, indicating a reduced degree of the level of peroxidation of guava fruit.

Different active oxygen scavengers function to maintain the balance of ROS metabolism and protect the membrane structure. SOD is an important antioxidant enzyme that is widespread in various organisms and catalyzes the dismutation of O^2−^ to hydrogen and molecular oxygen. CAT is a reactive oxygen scavenging enzyme that can degrade H_2_O_2_ into O_2_ and H_2_O to reduce the damage to plant tissues. GR is a flavoprotein oxidoreductase that is ubiquitous in eukaryotes and prokaryotes, and it is one of the important antioxidant enzymes for the active oxygen metabolism in plants. APX has some ability to remove active oxygen, which can effectively remove the H_2_O_2_ content in plants and effectively enhance the resistance of plants to external stresses.

As shown in Figure 3E, the activity of SOD decreased with fruit storage, and treatment with melatonin induced its activity, which was significantly higher than that of the control fruit. The activity of CAT increased first and reached a peak at day eight before gradually decreasing. Treatment with melatonin delayed the increase, resulting in much higher levels of activity compared with the control group (Figure 3F). Treatment with melatonin delayed the increase in the activity of GR during earlier storage and decreased during later storage, thus, maintaining higher GR activity during this period (Figure 3G). Melatonin also enhanced the activity of APX, which was higher compared with the control fruit during storage (Figure 3H).

### 3.4. Effects of Melatonin Treatment on the Content of Defense-Related Compounds

**Figure 4:**
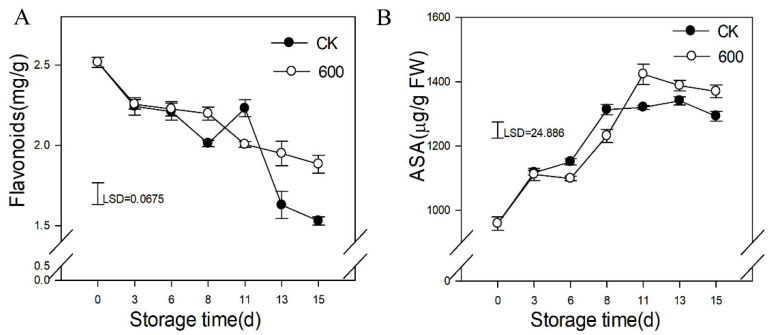
Effects of melatonin treatment on the content of defense-related substance in guava fruit. (**A**) the effect of melatonin treatment on flavonoids content; (**B**) the effect of melatonin treatment on ASA content. Each data point represents the mean ± S.D. (n = 3). Least significant differences (LSDs) were calculated to compare significant effects at the 5% level.CK: control group; 600: 600 μmol·L^−1^ melatonin treatment group.

Flavonoids are important secondary metabolites in plants. They have strong antioxidant activity and affect the color and flavor of the fruit. Flavonoids are a class of active substances that act as antioxidants and scavenge free radicals. As shown in Figure 4, the content of total flavonoids decreased with fruit storage, and treatment with melatonin reduced the decrease and maintained higher flavonoid contents than the control fruit during the later period of storage (Figure 4A).

The content of ASA increased with fruit ripening, and treatment with melatonin induced the accumulation of ASA during the later period of storage (Figure 4B). 

### 3.5. Effects of Melatonin Treatment on the Activities of Enzymes in the Phenylpropanoid Pathway

**Figure 5:**
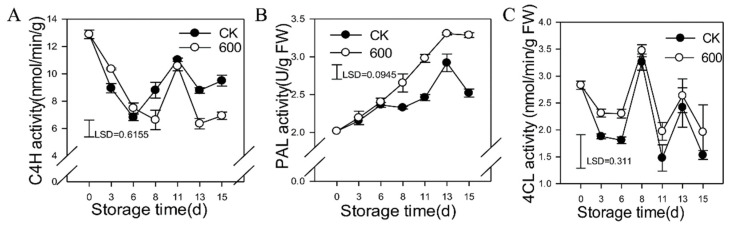
Effects of melatonin treatment on enzyme activities of the phenylpropanoid pathway in guava fruit. (**A**–**C**), the effect of melatonin treatment on the activities of C4H (**A**); PAL (**B**) and 4CL (**C**). Each data point represents the mean ± S.D. (n = 3). Least significant differences (LSDs) were calculated to compare significant effects at the 5% level. CK: control group; 600: 600 μmol·L^−1^ melatonin treatment group.

C4H is a key enzyme in the phenylpropanoid metabolic pathway, which can effectively regulate the synthesis of corresponding flavonoids and other metabolites. As shown in Figure 5A, the C4H activity generally decreased with fruit ripening and increased from days six to eleven. However, it decreased slightly during the later storage period (Figure 5A). Treatment with melatonin enhanced the activity of C4H on day three but reduced its activity during later storage (Figure 4A). Phenylalanine ammonia lyase (PAL) is one of the rate-limiting enzymes that plays critical roles in plant resistance. The activity of PAL increased gradually as the fruit ripened, and treatment with melatonin induced its activity. Significantly higher levels of PAL were observed in fruit treated with melatonin than the control during the later storage (Figure 5 B). 4CL is one of the key enzymes in lignin synthesis and catalyzes cinnamic acid to produce corresponding cinnamic acid-CoA esters, which are primarily found in higher plants, yeasts and fungi. The activity of 4CL had a trend of fluctuating–decreasing–increasing–decreasing–increasing. Treatment with melatonin induced its activity at some level (Figure 5C). 

### 3.6. Effects of Melatonin on the Activities of Lipid Metabolic Enzymes 

**Figure 6:**
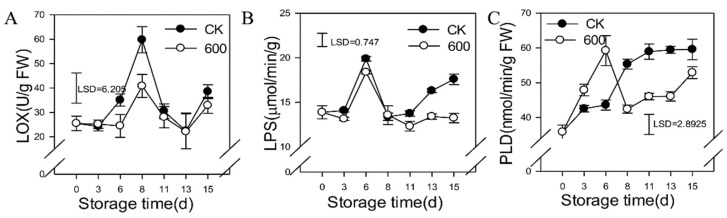
Effect of melatonin treatment on the activities of lipid metabolism enzymes in guava fruit. (**A**–**C**), the effect of melatonin treatment on the activities of LOX (**A**); LPS (**B**); PLD (**C**). Each data point represents the mean ± S.D. (n = 3). Least significant differences (LSDs) were calculated to compare significant effects at the 5% level. CK: control group; 600: 600 μmol·L^−1^ melatonin treatment group.

By catalyzing the oxidation reaction of unsaturated fatty acids, LOX can lead to membrane lipid peroxidation and play an important role in the maturation, senescence and adversity of stress in organisms. The activity of LOX increased gradually as the fruit ripened and reached its peak at day eight before decreasing. Treatment with melatonin reduced the activity of LOX, which was significantly lower than that of the control fruit during the first 11 days of storage (Figure 6A). LPS, also known as glyceride hydrolase, is widely present in a variety of organisms and can catalyzes the hydrolysis of triglycerides to produce fatty acids and glycerol. As shown in Figure 6B, the activity of LPS increased and reached a peak at day six, decreased rapidly and then increased slightly during the later period of storage. Treatment with melatonin severely repressed the activity of LPS during this period of storage (Figure 6B). The activity of PLD increased as the fruit ripened, and treatment with melatonin induced its activity during the first six days but severely inhibited its activity during later storage (Figure 6C).

### 3.7. Effects of Melatonin Treatment on the Activities of Enzymes Related to Resistance

**Figure 7:**
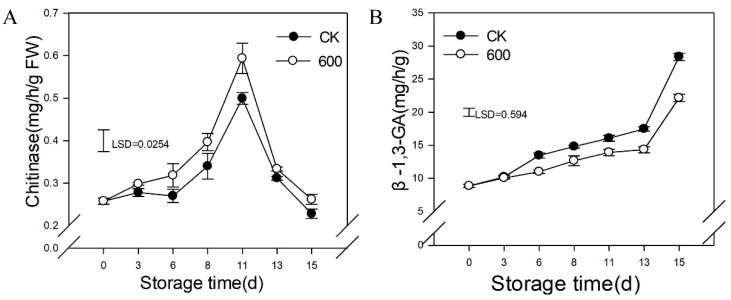
Effect of melatonin treatment on the activities resistance related enzymes in guava fruit. (**A**,**B**) the effect of melatonin treatment on the activities of CHI (**A**); β-1,3-GA (**B**). Each data point represents the mean ± S.D. (n = 3). Least significant differences (LSDs) were calculated to compare significant effects at the 5% level. CK: control group; 600: 600 μmol·L^−1^ melatonin treatment group.

CHI is a defense-related enzyme that is found widely in animals and plants. As shown in Figure 7A, the activity of CHI increased with fruit ripening and reached its peak on day 11 and then decreased during later storage (Figure 7A). Treatment with melatonin induced the activity of CHI, which had higher activity compared with the control group. The activity of β-1,3-GA increased gradually as the fruit ripened. Melatonin repressed the activity of β-1,3-GA during storage (Figure 7B).

## 4. Discussion

Melatonin is an important signaling molecule that functions as a master regulator in various developmental stages of plants and their adaptation to the environment [34,35]. Increasing numbers of studies have proven that the application of exogenous melatonin can effectively prolong storage and shelf life and maintain fruit quality [23,24,25,26,27]. A few studies also showed that the exogenous application of melatonin could accelerate the ripening of fruit, such as tomatoes. Treatment with melatonin substantially enhanced the accumulation of the content of lycopene and the production of ethylene in tomatoes, resulting in accelerated fruit ripening and an improvement in the quality of appearance of the tomato fruit [18]. Exogenous melatonin treatment on young grapes also significantly promoted the growth and increased the endogenous melatonin content of the fruit, significantly increasing the content of total anthocyanins, fruit total soluble solids and promoting the ripening of grapes [36]. This study demonstrates that treatment with melatonin could effectively delay the softening of guava fruit, which is consistent with most of the studies so far. Additionally, treatment with melatonin significantly reduces the incidence of anthracnose in guava fruit. Other studies also showed that the exogenous application of melatonin can significantly enhance the disease resistance and reduces the occurrence of decay of different fruit during storage by improving the antioxidant and defense systems of the fruit, such as peaches [26], strawberries [29], kiwifruits [30] and plums [37]. Therefore, as a safe and nontoxic substance, melatonin has a practical potential for application in postharvest guava fruit. Actually, the effects of melatonin are dose-dependent [35]. It was reported that 100 μM melatonin treatment could promote the ripening of grape fruit, while 0.1 and 1.0 μM melatonin showed no significant effect [38]. Melatonin application before flowering could delay the flowering of apple trees in a dose-dependent manner. The increased melatonin levels at a suitable range also resulted in more flowering, but unsuitably high concentrations would repress flowering [16]. The present work also showed that the effect of melatonin on fruit ripening is dose-dependent. Hence, the optimized concentration should be identified during application.

Fruit ripening often concomitantly proceeds with the accumulation of ROS, which function as the accelerator of the ripening process by adversely oxidizing membrane lipids, structural proteins and nucleic acids [39]. Fruit have evolved efficient non-enzymatic and enzymatic antioxidative systems to scavenge ROS and protect against oxidative damage [40]. The antioxidant enzymes, including CAT, APX, polyphenol oxidase (PPO), peroxidase (POD), dehydroascorbate reductase, SOD, GR and GPX, are important for ROS homeostasis in fruit [14]. The non-enzymatic antioxidants include ascorbic acid (AsA), flavonoids, anthocyanins, phenolics, carotenoids, dehydroascorbic acid and glutathione (GSH) [14,41]. Melatonin works as an excellent antioxidant molecule in plants. The application of exogenous melatonin can effectively reduce the accumulation of ROS in different fruits by inducing antioxidant systems, including the enzymatic antioxidants and non-enzymatic antioxidants, and then delay the process of fruit ripening [22,42,43]. For example, treatment with melatonin significantly enhanced the antioxidant capacity by increasing the total phenolic content, the scavenging ability of 1,1-diphenyl-2-picrylhydrazyl (DPPH) and 2,2′-Azinobis(3-ethylbenzothiazoline-6-sulfonic acid Ammonium Salt) (ABTS) and the activities of PAL and CHS, reducing the contents of MDA and H_2_O_2_ and decreasing ascorbic acid in fresh-cut pear fruit, resulting in reduced browning and microbial growth [44]. In strawberry fruit, treatment with exogenous melatonin significantly reduced the contents of H_2_O_2_ and MDA but enhanced the accumulation of total phenolics and flavonoids, resulting in an improved antioxidant capacity and a delay in fruit ripening [45]. In peach fruit, treatment with melatonin effectively increased the activities of CAT, SOD, APX and POD and reduced the content of MDA, O^2−^ and hydrogen peroxide, maintained the integrity of membranes and resulted in delayed postharvest senescence [26]. The application of melatonin also significantly increased the activities of CAT, SOD and POD in apple fruits, improving the antioxidant capacity of the fruit and maintaining its quality [22]. Non-enzymatic antioxidants, such as phenolics, GSH, AsA, flavonoids and anthocyanins, were also effectively induced by melatonin treatment [14,35,37]. The exogenous application of melatonin increased the polyphenol content and antioxidant activities in grapes [46]. This treatment also maintained the nutritional quality and reduced the decay of postharvest strawberry fruit by enhancing the contents of total phenolics, the accumulation of anthocyanins and γ-aminobutyric acid transaminase (GABA) shunt activity [29]. The exogenous application of melatonin increased the amount of phenolic compounds by upregulating the expression of related genes, which led to a delay in fruit senescence and deterioration in the quality of jujube fruit [47]. In this study, melatonin also effectively enhanced enzymatic antioxidants, such as the activities of SOD, GR, CAT and APX, and the content of non-enzymatic antioxidants, such as the total flavonoids, TP and ASA, and improving the T-AOC of the fruit, resulting in a reduction in the content of H_2_O_2_, O^2−^ and MDA. All these results proved that melatonin serves as an effective ROS scavenger to protect fruit from oxidative damage and maintain their quality.

Postharvest decays caused by diseases are a major cause of losses during the postharvest period. Most postharvest diseases could be controlled effectively by chemical fungicide treatments, but their use is strictly restricted owing to the increased enhancement of food safety awareness. Melatonin, a safe and nontoxic substance to humans and plants, functions as an environmentally friendly treatment that may be an alternative to fungicide applications and has shown its potential in postharvest preservation. A large number of studies have proven that melatonin can effectively reduce the postharvest disease incidence of various fruit, including bananas, apples, litchis, grapes, peaches, strawberries, plums and tomatoes [14,28]. The application of exogenous melatonin induced resistance to *Peronophythora litchii* in litchis by enhancing the activities of enzyme antioxidants, including PAL, C4H and 4CL, and increasing the accumulation of non-enzyme antioxidants, including phenolics and flavonoids [28]. Melatonin combined with *Meyerozyma guilliermondii* Y-1 effectively enhanced defense-related enzyme activities, including SOD, CAT, POD, PAL, and PPO, as well as increasing the content of non-enzyme antioxidants, such as the contents of TP and lignin, and the total antioxidant capacity (T-AOC), resulting in reduced disease incidence in apple fruit [48]. This study also showed that treatment with melatonin effectively reduces the incidence of anthracnose on fruit, which may result from an enhancement of the enzymatic antioxidants and non-enzymatic antioxidants, and the enhanced phenylpropanoid pathways, as well as an enhancement of the defense-related enzyme activities, including CHI. Some studies have shown that melatonin exhibited antimicrobial activities against different fruit pathogens in vitro and in vivo. In tomatoes, melatonin treatment effectively suppressed the growth of *Phytophthora infestans* in vitro and reduced the late blight symptoms of tomatoes in vivo [49]. Exogenous treatment with melatonin also induced the accumulation of arecoline and modulated the contents of ROS and levels of hormones, including ABA, JA, ethylene, and SA, as well as glycolytic activity in areca (*Arecha catechu*) fruit, resulting in improved plant disease resistance against *C. kahawae* and delayed postharvest physiological deterioration [50]. Melatonin can effectively induce the phenylpropanoid pathway and enhance the intermediate pathway for the synthesis of disease resistance-related phenolics in various fruit species. For example, in cherry tomato fruit, the application of melatonin effectively enhanced the accumulation of total phenolics, flavonoids and lignin by increasing the enzyme activities of the phenylpropanoid pathway, including 4CL, PAL and POD [21]. In tomatoes, melatonin treatment effectively reduced the development of the development of the virulent pathogen *Botrytis cinerea* by increasing the amounts of the endogenous resistance hormones SA, methyl jasmonate (MeJA) and melatonin, inducing an ROS burst, and enhancing the activities of the resistance enzymes of CHI and GLU and the phenylpropanoid pathway [21,51]. Melatonin treatment can also reduce the amount of pathogen infection by inducing the synthesis of cell walls, lipids and waxes in banana peels [19]. However, a few reports showed that melatonin could enhance disease. For example, melatonin treatment decreases the resistance of citrus fruit to postharvest green mold by reducing the level of defense-related ROS [52]. Strikingly, the effect of melatonin on fruit quality and postharvest disease could be owing to the signal-crossing talk in fruit and vegetables. There is widespread signal-crossing talk after melatonin treatment. Melatonin affects the signal molecules, such as ethylene, IAA, ABA, SA, GA, and NO among others, as well as endogenous melatonin in plants, to regulate fruit ripening and stress responses [14]. The application of exogenous melatonin can also induce the accumulation of endogenous melatonin during the fruit ripening process and affect fruit quality and shelf life of such fruit as grapes [53], pears [25], sweet cherries [23] and bananas [24]. Thus, the effects of melatonin treatment can be dose-dependent or species-dependent.

Fruit respiration is important for fruit physiology during ripening process. Melatonin treatment effectively reduced the elevation of the respiratory rate in bananas [19], pears [25], mango fruits [27], as well as altered the secondary metabolism pathways, resulting in the delayed ripening process. Preharvest spray with melatonin depressed fruit respiration after harvest while melatonin induced respiratory activity following cold storage [54]. It indicated that the sweet cherry response to melatonin treatment involved a cold-dependent activation of the respiration and up-regulation of TCA cycle-related genes [54]. It seems that cold storage may cause stress to sweet cherry fruit and give rise to ROS. Melatonin may enhance the respiration and improve the energy supply to maintain fruit quality [28]. Importantly, melatonin induced phenolic compound accumulation and related genes expression, which help to improve fruit quality and cold tolerance [54]. In the present work, it was established that melatonin repressed fruit respiration rates during the early storage period, but increased respiration during later storage, which corresponded to the enhanced total flavonoids and ascorbic acid accumulation. The increased respiration may contribute to the accumulation of secondary metabolites, helping the disease response. Melatonin treatment also maintained fruit firmness and reduced the change in fruit color, retarding fruit ripening and senescence.

## 5. Conclusions

**Figure 8:**
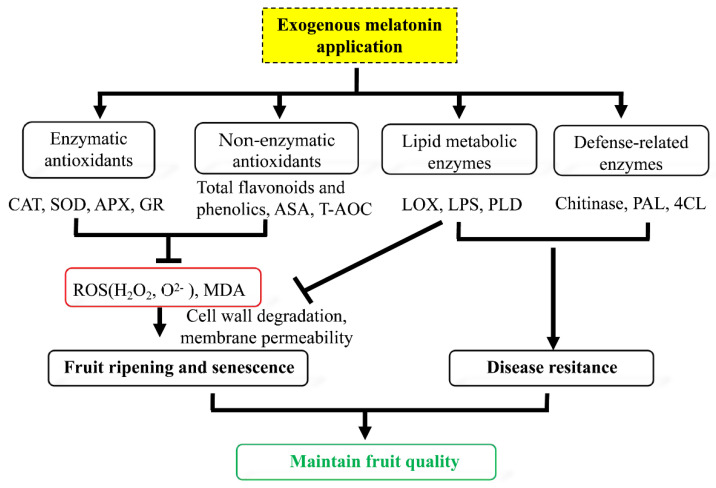
Proposed roles of exogenous melatonin treatment to maintain postharvest guava fruit quality. ROS: reactive oxygen species; MDA: malondialdehyde; CAT: catalase; SOD: superoxide dismutase; GR: glutathione reductase; APX: ascorbate peroxidase; ASA: ascorbic acid; 4CL: 4-coumaric acid: coenzyme A ligase; PAL: phenylalanine ammonia lyase; LPS: lipase; LOX: lipoxygenase; PLD: phospholipase D.

As shown in Figure 8, in this study we showed that treatment with melatonin effectively enhanced the antioxidant capacity, the content of total flavonoids and ascorbic acid and the activity of phenylpropanoid pathway enzymes, such as PAL and 4CL, and reduced the oxidative damage of guava fruit. Melatonin treatment also enhanced the activities of defense-related enzymes, including CHI, and repressed the activities of LPS, LOX and PLD related to lipid metabolism. All these results showed that treatment with exogenous melatonin can maintain fruit quality and enhance disease resistance by improving the antioxidant and defense systems.

## Data Availability

The data presented in this study are available on request from the corresponding author.

## Supplementary Materials

The following supporting information can be downloaded at: https://www.mdpi.com/article/10.3390/foods11030262/s1, Figure S1: Overview of physiological index changes presented in Figure 1 and Figure 2 of guava fruit in response to melatonin treatments during storage; Table S1: Dataset for all figures in present work.

## References

[1] Singh S.P. (2011). Guava (Psidium guajava L.). Postharvest Biology and Technology of Tropical and Subtropical Fruits: Cocona to Mango.

[2] McCook-Russell K.P., Nair M.G., Facey P.C., Bowen-Forbes C.S. (2012). Nutritional and nutraceutical comparison of Jamaican Psidium cattleianum (strawberry guava) and Psidium guajava (common guava) fruits. Food Chem..

[3] Murmu S.B., Mishra H.N. (2018). Post-harvest shelf-life of banana and guava: Mechanisms of common degradation problems and emerging counteracting strategies. Innov. Food Sci. Emerg. Technol..

[4] Singh S.P., Pal R.K. (2008). Response of climacteric-type guava (Psidium guajava L.) to postharvest treatment with 1-MCP. Postharvest Biol. Technol..

[5] González-Aguilar G.A., Tiznado-Hernández M.E., Zavaleta-Gatica R., Martínez-Téllez M.A. (2004). Methyl jasmonate treatments reduce chilling injury and activate the defense response of guava fruits. Biochem. Biophys. Res. Commun..

[6] Zakaria L. (2021). Diversity of Colletotrichum Species Associated with Anthracnose Disease in Tropical Fruit Crops—A Review. Agriculture.

[7] Oliveira P.D.L., de Oliveira K.Á.R., Dos Santos Vieira W.A., Câmara M.P.S., de Souza E.L. (2018). Control of anthracnose caused by *Colletotrichum* species in guava, mango and papaya using synergistic combinations of chitosan and *Cymbopogon citratus* (D.C. ex Nees) Stapf. essential oil. Int. J. Food Microbiol..

[8] Alba-Jiménez J.E., Benito-Bautista P., Nava G.M., Rivera-Pastrana D.M., Vázquez-Barrios M.E., Mercado-Silva E.M. (2018). Chilling injury is associated with changes in microsomal membrane lipids in guava fruit (*Psidium guajava* L.) and the use of controlled atmospheres reduce these effects. Sci. Hortic..

[9] Vishwasrao C., Ananthanarayan L. (2016). Postharvest shelf-life extension of pink guavas (*Psidium guajava* L.) using HPMC-based edible surface coatings. J. Food Sci. Technol..

[10] Lo’ay A.A., Taher M.A. (2018). Influence of edible coatings chitosan/PVP blending with salicylic acid on biochemical fruit skin browning incidence and shelf life of guava fruits cv. ‘Banati.’. Sci. Hortic..

[11] Azam M., Hameed L., Qadri R., Ejaz S., Ghani M.A. (2021). Postharvest ascorbic acid application maintained physiological and antioxidant responses of Guava (*Psidium guajava* L.) at ambient storage. Food Sci. Technol..

[12] Sahu S.K., Barman K., Singh A.K. (2020). Nitric oxide application for postharvest quality retention of guava fruits. Acta Physiol. Plant..

[13] Botelho R.V., Souza N., Peres N. (2000). Effect of the postharvest treatment with calcium chloride by the temperature differential method on the control of Colletotrichum gloeosporioides in guavas “Branca de Kumagaii”. Summa Phytopathol..

[14] Ze Y., Gao H., Li T., Yang B., Jiang Y. (2021). Insights into the roles of melatonin in maintaining quality and extending shelf life of postharvest fruits. Trends Food Sci. Technol..

[15] Zhang N., Zhang H.-J., Sun Q.-Q., Cao Y.-Y., Li X., Zhao B., Wu P., Guo Y.-D. (2017). Proteomic analysis reveals a role of melatonin in promoting cucumber seed germination under high salinity by regulating energy production. Sci. Rep..

[16] Zhang H., Wang L., Shi K., Shan D., Zhu Y., Wang C., Bai Y., Yan T., Zheng X., Kong J. (2019). Apple tree flowering is mediated by low level of melatonin under the regulation of seasonal light signal. J. Pineal Res..

[17] Wang C., Yin L.-Y., Shi X.-Y., Xiao H., Kang K., Liu X.-Y., Zhan J.-C., Huang W.-D. (2016). Effect of Cultivar, Temperature, and Environmental Conditions on the Dynamic Change of Melatonin in Mulberry Fruit Development and Wine Fermentation. J. Food Sci..

[18] Sun Q., Zhang N., Wang J., Zhang H., Li D., Shi J., Li R., Weeda S., Zhao B., Ren S. (2015). Melatonin promotes ripening and improves quality of tomato fruit during postharvest life. J. Exp. Bot..

[19] Li T., Wu Q., Zhu H., Zhou Y., Jiang Y., Gao H., Yun Z. (2019). Comparative transcriptomic and metabolic analysis reveals the effect of melatonin on delaying anthracnose incidence upon postharvest banana fruit peel. BMC Plant Biol..

[20] Cao S., Bian K., Shi L., Chung H.-H., Chen W., Yang Z. (2018). Role of Melatonin in Cell-Wall Disassembly and Chilling Tolerance in Cold-Stored Peach Fruit. J. Agric. Food Chem..

[21] Li S., Xu Y., Bi Y., Zhang B., Shen S., Jiang T., Zheng X. (2019). Melatonin treatment inhibits gray mold and induces disease resistance in cherry tomato fruit during postharvest. Postharvest Biol. Technol..

[22] Onik J.C., Wai S.C., Li A., Lin Q., Sun Q., Wang Z., Duan Y. (2021). Melatonin treatment reduces ethylene production and maintains fruit quality in apple during postharvest storage. Food Chem..

[23] Tijero V., Muñoz P., Munné-Bosch S. (2019). Melatonin as an inhibitor of sweet cherries ripening in orchard trees. Plant Physiol. Biochem..

[24] Hu W., Yang H., Tie W., Yan Y., Ding Z., Liu Y., Wu C., Wang J., Reiter R.J., Tan D.-X. (2017). Natural Variation in Banana Varieties Highlights the Role of Melatonin in Postharvest Ripening and Quality. J. Agric. Food Chem..

[25] Liu J., Yang J., Zhang H., Cong L., Zhai R., Yang C., Wang Z., Ma F., Xu L. (2019). Melatonin Inhibits Ethylene Synthesis via Nitric Oxide Regulation To Delay Postharvest Senescence in Pears. J. Agric. Food Chem..

[26] Gao H., Zhang Z.K., Chai H.K., Cheng N., Yang Y., Wang D.N., Yang T., Cao W. (2016). Melatonin treatment delays postharvest senescence and regulates reactive oxygen species metabolism in peach fruit. Postharvest Biol. Technol..

[27] Liu S., Huang H., Huber D.J., Pan Y., Shi X., Zhang Z. (2020). Delay of ripening and softening in ‘Guifei’ mango fruit by postharvest application of melatonin. Postharvest Biol. Technol..

[28] Zhang Z., Wang T., Liu G., Hu M., Yun Z., Duan X., Cai K., Jiang G. (2021). Inhibition of downy blight and enhancement of resistance in litchi fruit by postharvest application of melatonin. Food Chem..

[29] Aghdam M.S., Fard J.R. (2017). Melatonin treatment attenuates postharvest decay and maintains nutritional quality of strawberry fruits (*Fragaria*×*anannasa* cv. Selva) by enhancing GABA shunt activity. Food Chem..

[30] Hu M., Li J., Rao J. (2018). Effect of Melatonin on Ripening and Senescence of Postharvest Kiwifruits. Food Sci..

[31] Zhu X., Ye L., Ding X., Gao Q., Xiao S., Tan Q., Huang J., Chen W., Li X. (2019). Transcriptomic analysis reveals key factors in fruit ripening and rubbery texture caused by 1-MCP in papaya. BMC Plant Biol..

[32] Li X., Zhu X., Mao J., Zou Y., Fu D., Chen W., Lu W. (2013). Isolation and characterization of ethylene response factor family genes during development, ethylene regulation and stress treatments in papaya fruit. Plant Physiol. Biochem..

[33] Pandey R.R., Arora D.K., Dubey R.C. (1997). Effect of environmental conditions and inoculum density on infection of guava fruits by Colletotrichum glososporioides. Mycopathologia.

[34] Arnao M.B., Hernández-Ruiz J. (2019). Melatonin: A New Plant Hormone and/or a Plant Master Regulator?. Trends Plant Sci..

[35] Wu X., Ren J., Huang X., Zheng X., Tian Y., Shi L., Dong P., Li Z. (2021). Melatonin: Biosynthesis, content, and function in horticultural plants and potential application. Sci. Hortic..

[36] Yang M., Wang L., Belwal T., Zhang X., Lu H., Chen C., Li L. (2020). Exogenous Melatonin and Abscisic Acid Expedite the Flavonoids Biosynthesis in Grape Berry of Vitis vinifera cv. Kyoho. Molecules.

[37] Bal E. (2019). Physicochemical changes in ‘Santa Rosa’ plum fruit treated with melatonin during cold storage. J. Food Meas. Charact..

[38] Xu L., Yue Q., Xiang G., Bian F., Yao Y. (2018). Melatonin promotes ripening of grape berry via increasing the levels of ABA, H_2_O_2_, and particularly ethylene. Hortic. Res..

[39] Corpas F.J., Freschi L., Rodríguez-Ruiz M., Mioto P.T., González-Gordo S., Palma J.M. (2018). Nitro-oxidative metabolism during fruit ripening. J. Exp. Bot..

[40] Jiang Y., Duan X., Joyce D., Zhang Z., Li J. (2004). Advances in understanding of enzymatic browning in harvested litchi fruit. Food Chem..

[41] Mirshekari A., Madani B., Yahia E.M., Golding J.B., Vand S.H. (2020). Postharvest melatonin treatment reduces chilling injury in sapota fruit. J. Sci. Food Agric..

[42] Wang S.-Y., Shi X.-C., Wang R., Wang H.-L., Liu F., Laborda P. (2020). Melatonin in fruit production and postharvest preservation: A review. Food Chem..

[43] Liu J., Liu H., Wu T., Zhai R., Yang C., Wang Z., Ma F., Xu L. (2019). Effects of Melatonin Treatment of Postharvest Pear Fruit on Aromatic Volatile Biosynthesis. Molecules.

[44] Zheng H., Liu W., Liu S., Liu C., Zheng L. (2019). Effects of melatonin treatment on the enzymatic browning and nutritional quality of fresh-cut pear fruit. Food Chem..

[45] Liu C., Zheng H., Sheng K., Liu W., Zheng L. (2018). Effects of melatonin treatment on the postharvest quality of strawberry fruit. Postharvest Biol. Technol..

[46] Xu L., Yue Q., Bian F., Sun H., Zhai H., Yao Y. (2017). Melatonin Enhances Phenolics Accumulation Partially via Ethylene Signaling and Resulted in High Antioxidant Capacity in Grape Berries. Front. Plant Sci..

[47] Wang L., Luo Z., Ban Z., Jiang N., Yang M., Li L. (2021). Role of exogenous melatonin involved in phenolic metabolism of Zizyphus jujuba fruit. Food Chem..

[48] Sun C., Huang Y., Lian S., Saleem M., Li B., Wang C. (2021). Improving the biocontrol efficacy of Meyerozyma guilliermondii Y-1 with melatonin against postharvest gray mold in apple fruit. Postharvest Biol. Technol..

[49] Zhang S., Zheng X., Reiter R.J., Feng S., Wang Y., Liu S., Jin L., Li Z., Datla R., Ren M. (2017). Melatonin Attenuates Potato Late Blight by Disrupting Cell Growth, Stress Tolerance, Fungicide Susceptibility and Homeostasis of Gene Expression in Phytophthora infestans. Front. Plant Sci..

[50] Yin X., Wei Y., Song W., Zhang H., Liu G., Chen Y., Li L.-Z., Alolga R.N., Ma G., Reiter R.J. (2020). Melatonin as an inducer of arecoline and their coordinated roles in anti-oxidative activity and immune responses. Food Funct..

[51] Liu C., Chen L., Zhao R., Li R., Zhang S., Yu W., Sheng J., Shen L. (2019). Melatonin Induces Disease Resistance to Botrytis cinerea in Tomato Fruit by Activating Jasmonic Acid Signaling Pathway. J. Agric. Food Chem..

[52] Lin Y., Fan L., Xia X., Wang Z., Yin Y., Cheng Y., Li Z. (2019). Melatonin decreases resistance to postharvest green mold on citrus fruit by scavenging defense-related reactive oxygen species. Postharvest Biol. Technol..

[53] Wang L., Luo Z., Yang M., Li D., Qi M., Xu Y., Abdelshafy A.M., Ban Z., Wang F., Li L. (2020). Role of exogenous melatonin in table grapes: First evidence on contribution to the phenolics-oriented response. Food Chem..

[54] Michailidis M., Tanou G., Sarrou E., Karagiannis E., Ganopoulos I., Martens S., Molassiotis A. (2021). Pre- and Post-harvest Melatonin Application Boosted Phenolic Compounds Accumulation and Altered Respiratory Characters in Sweet Cherry Fruit. Front. Nutr..

